# Comparing undergraduate research experiences before, during, and after the COVID-19 quarantine: The successful adaptation of the BUILD PODER Summer JumpStart program

**DOI:** 10.1371/journal.pone.0295901

**Published:** 2023-12-28

**Authors:** Patricia Escobedo, Daniel Garcia, Liam Cascelli, Gabriela Chavira, Gilberto E. Flores, Jodi L. Constantine Brown, David Boyns, Andrew T. Ainsworth

**Affiliations:** 1 Department of Psychology, California State University, Northridge, Northridge, California, United States of America; 2 School of Education, University of California, Irvine, Irvine, California, United States of America; 3 Center for Assessment, Research, and Evaluation (CARE), California State University, Northridge, Northridge, California, United States of America; 4 Department of Biology, California State University, Northridge, Northridge, California, United States of America; 5 Department of Social Work, California State University, Northridge, Northridge, California, United States of America; 6 Department of Sociology, California State University, Northridge, Northridge, California, United States of America; University of Sharjah College of Health Sciences, UNITED ARAB EMIRATES

## Abstract

In March 2020, the COVID-19 pandemic forced many in person undergraduate research experiences (UREs) to pivot to remote online training. To investigate how the COVID-19 quarantine disrupted student URE outcomes over time, the current study examines Building Infrastructure Leading to Diversity (BUILD) Promoting Opportunities for Diversity in Education and Research (PODER) URE outcomes across different platforms (in-person, remote, and hybrid models) by comparing student survey data from 2019 to 2021. Participants consisted of three cohorts: 2019 (n = 26 students), 2020 (n = 33), 2021 (n = 34). The BUILD PODER Summer JumpStart program (SJS), which aims to increase diversity in Science, Technology, Engineering, and Mathematics (STEM) by recruiting mostly underrepresented students, was conducted in person in 2019, remotely in 2020 and using a hybrid model in 2021. All students completed an online survey on the first and last day of the four-week SJS program. We used one-way and mixed ANOVA models to analyze Cohort, Time (pre-test vs. post-test scores), and interaction of Cohort and Time for Research Self-Efficacy, Sense of Belonging, Mentor Relationship, Mentee Knowledge, Health, Stress, and Student Program Satisfaction measures. Despite the platform changes, student scores increased significantly over time for all measures. There was a significant main effect of Time for Research Self-Efficacy, Sense of Belonging, Mentor Relationship, Mentee Knowledge, Health Assessment, and Stress Management. Findings indicate that URE programs that are implemented remotely and using a hybrid format can provide students with experiences similar to in-person URE programs. In addition, remote UREs may provide added benefits compared to in-person programs. For instance, remote UREs could engage more historically minoritized students, who may experience barriers to access, such as work/family commitments, financial constraints, and geographic limitations.

## Introduction

Over the past few decades, the scientific community has made great efforts to diversify the scientific workforce [1, 2]. In particular, Black and Latinx students are less likely to pursue science, technology, engineering, and mathematics (STEM) majors and less likely to pursue STEM-related careers than their white or Asian counterparts [3]. To address these disparities, many institutions have developed initiatives to increase access to STEM education, which have increased the enrollment of minoritized students [4]. Yet despite some gains, students from ethnic-racial minoritized communities remain less likely to persist in STEM postsecondary majors [3]. High-impact practices (HIPs) positively affect student retention, success, and graduation [5, 6]. HIPs are active learning approaches that foster deep learning by promoting student engagement and positively impacting historically underserved students. Undergraduate research experiences (URE) are powerful HIPs that unequivocally show improvements in student retention and success [2, 5].

Short-term (i.e., summer) and longer-term (i.e., academic year) undergraduate research experiences (UREs) are traditionally delivered in person [7]. This format has effectively retained undergraduates in STEM through their graduate degrees and careers, especially students from minoritized communities [8, 9]. In March 2020, the COVID-19 pandemic forced UREs across the U.S. to cancel their summer programs, with few programs pivoting to remote online experiences for summer training [7, 10–12]. The pandemic allowed programs to examine the efficacy of online/remote undergraduate research training [11–13]. While some research has compared pre-pandemic to pandemic program implementation [14], to our knowledge, no studies have examined programmatic outcomes across different methods of implementation (i.e., in-person pre-pandemic, remote during COVID-19 quarantine, hybrid after some restrictions were lifted) to investigate the extent to which the quarantine created disruptions in student outcomes. Moreover, examining these student outcomes across platforms is important to demonstrate if the core objectives of traditional in-person UREs can be accomplished virtually, especially when unforeseen disruptions occur.

### Disparities in STEM majors and careers

During the past 30 years, U.S. federal agencies increased efforts to diversify the STEM workforce, which yielded successful equity initiatives and research training opportunities for underrepresented scholars [2, 15]. However, disparities persist. Black, Latinx, American Indian/Alaska Natives, and Hawaiians/Pacific Islanders comprise 32% of the US population, yet they are historically minoritized; that is, they remain underrepresented in the sciences compared to Asians and Whites [16]. For instance, in 2018, only 22.5% of STEM bachelor’s degrees were awarded to historically minoritized groups [3]. This is concerning given that in 2011, the National Academy of Sciences (NAS) reported that most of the growth in new jobs will require science and technology skills [17]. To create a diverse STEM workforce, NAS recommended doubling the number of historically minoritized undergraduates earning STEM degrees [17]. To accomplish this goal, there is an urgent need to target and train students from minoritized groups with the research skills and self-efficacy necessary to earn undergraduate and graduate STEM degrees.

### Undergraduate Research Experiences (UREs)

Interventions focused on increasing the number of students from minoritized groups earning undergraduate STEM degrees should offer students opportunities to participate in URE programs. Such programs include Building Infrastructure Leading to Diversity (BUILD) [18], Research Experiences for Undergraduates (REU) [19], Ronald E. McNair Scholars Program [20], Research Initiative for Scientific Enhancement (RISE) [2], Supporting Undergraduate Research Experiences (SURE) [14], the Meyerhoff Scholars Program [21], Biomedical Career Enrichment Programs (BCEPs), Einstein/Montefiore program and Woods Hole Partnership Education (PEP) [11, 22, 23]. UREs offer undergraduates exposure to research projects, faculty, and advanced peers who can provide access to professional connections and acquaint students with scientific norms [24–26]. For instance, URE faculty and peer support systems have been shown to promote gains among student participants in active learning, self-confidence, and pursuit of science careers [27, 28]. URE programs also provide spaces for demonstrating science discourse and practice, facilitating undergraduates’ scientific identities [29]. As a result, URE participants are better prepared to become science professionals and succeed in STEM programs than their peers [8, 30–32]. Moreover, UREs increase students’ critical thinking and communication skills than course-based research projects [31, 33–35].

Research on URE outcomes show there are positive effects on students’ intentions to enroll and actual enrollment in STEM graduate and professional programs [8–10, 19, 36, 37]. For example, Wilson and colleagues examined five NSF-funded UREs [19]. They found that URE participants were more likely to pursue a Ph.D. program and produce more presentations, publications, and awards than students who applied to the URE but did not participate. UREs provide undergraduates opportunities to develop scientific professional skills, explore possible career options, and refine post-undergraduate goals [26].

### Increasing ethnic-racial minoritized groups’ participation in STEM

Some of the long-term goals of URE programs is to help increase the diversity of the scientific workforce in the U.S. by broadening the pool of student participants, improving retention, and increasing matriculation into competitive graduate programs [38, 39]. UREs typically provide undergraduate training through a multi-institution consortium that includes several research partners and various pipeline partners [37, 40, 41]. Key features of these programs include mentor-driven practices to support undergraduate student researchers as well as training faculty in research pedagogy and mentoring techniques and providing faculty with resources for increasing their research productivity [19, 40–43].

Research shows that UREs aimed at increasing the number of underrepresented minoritized students in the sciences can improve students’ likelihood of enrolling in doctorate programs [37, 38, 44]. For example, a previous study examined 13 student cohorts of the Meyerhoff Scholarship Program, which focuses on increasing the number of ethnic-racial minoritized students who earn doctorate degrees and pursue research careers in STEM fields [38]. This study found that URE participation was associated with a significant increase in the probability of minoritized students pursuing a science or engineering doctorate [38]. Further, many UREs are designed to support minoritized undergraduates, are successful at helping these students graduate with a STEM degree [39, 45] and can facilitate intentions to pursue a scientific research career [15, 36, 41, 44]. In addition, UREs tailored for minoritized undergraduates help improve retention in STEM [19, 44, 46]. For minoritized students to persist in the sciences, UREs should consider incorporating a culturally-affirming curriculum that fosters the development of a science identity and interest in pursuing a scientific career [36]. In addition, UREs that focus on critical mentorship and asset-based curricula that affirm the strengths of student’s culture/ethnicity are being designed to recruit and train minoritized students [36, 47–49]. Yet, it wasn’t until 2017 that the National Institute on General Medical Sciences (NIGMS) required that all training grant applications state that program mentors will receive training in mentorship [50]. Similarly, in 2022, the Research Experiences for Undergraduates (REU), funded by the National Science Foundation, strongly encouraged their REUs to provide training for research mentors [51], as they previously only encouraged training for new research mentors [52].

### Differences in learning platform

Previous research exploring differences between the traditional classroom and remote or hybrid learning options has focused on student satisfaction [53] or grades [54] as outcomes, with few studies exploring student research self-efficacy or well-being. In a longitudinal study of social work graduate students’ research self-efficacy, Constantine Brown and Park found that student knowledge and research self-efficacy improved between pre-test and post-test on standardized measures and remained significantly improved at follow-up one year later, with no significant difference between online learners and traditional on-site students [55]. URE program designs remained largely traditional until the COVID-19 pandemic forced a rapid shift to remote online training [7, 10–12]. Three studies that measured student and program outcomes after training was completed, found that students reported the virtual program was a positive experience [11, 13], and students reported increased interest in pursuing biomedical careers, increases in research skills, positive relationship with mentors, and increased mental wellbeing after virtual program participation [12]. To date, no studies of URE have compared student outcomes by learning platform across different cohorts.

### BUILD PODER: Objectives and Summer JumpStart (SJS)

Founded in 2014 through a common fund from the National Institutes of Health (NIH), the Building Infrastructure Leading to Diversity (BUILD) initiative aims to increase the diversity of the research workforce by preparing undergraduate student researchers from diverse groups in preparation for graduate studies in biomedical sciences. The BUILD program based at California State University, Northridge program is known as BUILD Promoting Opportunities for Diversity in Education and Research (PODER) and is one of ten BUILD sites across the United States. BUILD PODER employs Critical Race Theory (CRT) and related theories rooted in a social justice perspective throughout the student training curriculum and takes community-based, holistic, and transdisciplinary approaches to identify and resolve social problems such as health disparities. For example, BUILD PODER students use the National Science Foundation’s National Center for Science and Engineering Statistics (NCSES) [56] to review data on diversity in STEM and track their own trajectories through higher education and into science careers to highlight the disparities in the STEM workforce. BUILD PODER also incorporates discussions of current real-world health disparities, such as unequal access to healthcare during the COVID-19 pandemic that resulted in higher cases of infection and deaths related to COVID-19 among low-income and ethnically minoritized communities, as well as discussions centered around historical accounts of ethical violations and mistrust of medical scientists. BUILD PODER extends Solórzano and Yosso’s critical race analytical approach [57] to research in education by using the theory’s tenets as guides in all aspects of the research training curriculum and programming for faculty and students. BUILD PODER also employs Yosso’s Cultural Community Wealth Model [58] as an asset-based approach to challenge traditional forms (e.g., wealth, education) of capital by valuing non-traditional forms of capital (e.g., language and cultural knowledge).

Newly accepted BUILD PODER students begin training by participating in a four-week intensive entry-to-research Summer JumpStart (SJS) program (see S1 Fig for student recruitment and program timeline). The SJS program is an important component of the BUILD PODER URE as the training program is designed to provide students with biomedical research skills. However, SJS is also designed to help students build positive mentee-mentor relationships, increase feelings of research self-efficacy, develop a sense of belonging, and increase overall well-being to help underrepresented students persist in STEM majors, earn STEM degrees, and enter the STEM workforce.

### Summer JumpStart objectives

#### Research Self-Efficacy

Self-efficacy is an individual’s belief about their ability to perform tasks or activities related to a specific goal or outcome [59]. Research self-efficacy, therefore, is a student’s self-perception of their ability to perform research-related tasks and activities [60]. Research on science self-efficacy indicates it is a predictor of a students’ persistence in science-related fields [61]. Research self-efficacy and research skills were found to predict students’ aspirations for research careers, and the effects of research skills are partially mediated through self-efficacy beliefs [62]. Similarly, Pajares argued that students’ research self-efficacy is a vital link between their acquisition of research skills and knowledge, and how they apply these skills [63]. Furthermore, studies found that UREs can positively impact a research self-efficacy among students, which included the ability to think and work like a scientist, understanding how science is conducted and the ability to complete research-related tasks (e.g., laboratory or fields work, analyzing and interpreting data, research presentations) [34, 64, 65].

#### Mentoring relationship

Faculty mentorship provides instrumental psychosocial, emotional, and networking functions for students [66–68]. A previous study found that research and mentoring experiences with faculty significantly improve students’ self-rated science efficacy [69]. Previous research also found an association between faculty mentoring, academic success, and persistence [70]. Research suggests mentoring is especially critical for minoritized students [49, 71–73]. For instance, in a study of 277 minoritized students, Castellanos and colleagues found that students who reported having a mentor had higher cultural fit (i.e., cultural congruity in combination with a perception of the university environment), more support (i.e., psychosocial support, and networking), and higher college and life satisfaction [72]. Furthermore, minoritized students’ perceptions of mentorship have been linked to increased retention [74], academic goal definition, and college adjustment [75].

#### Sense of Belonging and health

Students who report feeling a sense of community in the classroom are more likely to attend class, participate during class, and graduate from college [76–78]. Furthermore, a sense of community has been shown to correlate positively with a student’s likelihood to contribute during class discussions and negatively with a student’s anxiety in the classroom [79]. These factors are particularly salient among undergraduate, first-generation, and minoritized populations [80–82]. Previous research indicates that success in STEM relies on a sense of belonging, especially among minoritized students [11, 81]. Previous studies demonstrate that UREs can influence students’ feelings of connectedness and well-being [83, 84], and previous research also indicates that science identity is facilitated by a sense of community and affiliation [29].

Individuals have an innate need to belong to communities, motivating them to maintain lasting, positive relationships and strongly affecting emotional and cognitive functioning [85]. Failure to make connections with others can negatively affect mental health and behavior [85]. For the community to impact an individual student, the student must perceive themselves as part of that community [86]. Therefore, the present study investigates students’ sense of community within the summer URE program and its role in increasing positive student outcomes.

### Purpose

To develop effective program implementation and increase URE accessibility, especially when unexpected disruptions occur, the current study examined if student outcomes from the summer UREs differed significantly across formats (in-person, remote, or hybrid). The following research questions guided the study:

1. How does the URE program format impact changes in Research Self-Efficacy, Sense of Belonging, Mentor Relationship, Mentee Knowledge, Health, and Stress Management scores across time?
2. How does the URE program format impact ratings of Program Satisfaction among student participants?

## Methods

### Procedure

A total of 93 undergraduate students participated in the summer entry-to-research training program. The participants included 26 students in 2019, 33 in 2020, and 34 in 2021. To join BUILD PODER at California State University, Northridge (CSUN), individuals needed to be currently enrolled at one of four Pipeline Partners (PP) community colleges in the university’s service area or be a CSUN undergraduate student in their sophomore or junior year. The selection process consists of three steps: 1) students apply, 2) applications are screened for inclusionary criteria by a review panel, and 3) selected students are interviewed. BUILD PODER is a two to three-year program, depending on the coursework needed for graduation. All newly accepted BUILD PODER students complete the Summer JumpStart (SJS), a four-week intensive summer research experience held during the month of July that includes training on: research skill-building, building positive mentor-mentee relationships, research ethics, community-building, CRT principles and maintaining physical and mental health. Upon completion of SJS, students enter their first year in BUILD PODER.

Since its inception in 2015, BUILD PODER SJS has been conducted in person on the university campus, and since 2017, all participants have been provided complimentary on-campus housing. SJS participants completed community-building activities, attended workshops, research presentations, and field trips, and worked on projects in their mentor’s research labs. However, in 2020 all BUILD PODER SJS programming was moved entirely online in response to the COVID-19 pandemic. To complete lab work and other hands-on training or activities during remote programming, students picked up complimentary supplies provided by program staff from the university as needed (curb-side pickup), or it was mailed to participants if they were not within driving distance. In 2021, SJS began as an in-person program, with five students beginning the training remotely due to not being fully vaccinated for COVID-19 per BUILD PODER’s policy. However, all students were in-person by week three of the 2021 SJS program.

The current study used SJS data from 2019 to 2021. Trained research investigators administered all pre-test and post-test surveys. Only students who provided informed consent were given a link to complete an online survey on a personal device (e.g., laptop, tablet, or cell phone). Pre-test surveys were administered on the first day of SJS, and post-test surveys were administered four weeks later on the last day of SJS. In 2019, all surveys were administered on the CSUN campus, and in 2020 all surveys were administered remotely. In 2021, the pre-test survey was administered remotely, but the post-test survey was completed on the university campus. All students completed the same survey. The full survey took around 60 minutes to complete. The CSUN Committee for the Protection of Human Subjects approved all procedures.

### Participant characteristics

The survey asked SJS participants to provide demographic information, including gender, first-generation college status, and Federal Pell Grant status. The U.S. Department of Education defines Pell Grants as awards to first-time undergraduate students with “exceptional financial need,” and award status is often used as an indication of low-income status [87]. Additional demographic information, such as ethnicity, was obtained from program records. Ethnicity was coded as Asian, Black, Latinx, White, and Other (multi-ethnic). First-generation college status and Pell Grant recipient status were coded as dichotomous variables (1 = “yes,” 0 = “no”).

### Measures

#### Research Self-Efficacy

Research Self-Efficacy was assessed using a 9-item scale. The questions were adapted from the 30-item Undergraduate Research Student Self-Assessment (URSSA) survey [65]. The nine items used in this survey focused on personal gains related to research work. Students were asked to rate their level of confidence or comfort level with various research activities, which included “comfort in discussing scientific concepts with others,” “confidence in my ability to do well in future courses,” and “confidence in engaging in real-world science research.” Responses were coded 1 = “none”, 2 = “little”, 3 = “moderate”, 4 = “quite a bit”, and 5 = “a lot” (see S1 Appendix all measure items and response options). Composite scores were created by averaging item responses to form a single composite score. Composite scores were calculated for pre-test items and post-test items. Cronbach’s alpha [α] statistics were calculated for pre-test items (α = 0.879) and post-test items (α = 0.906).

#### Sense of Belonging

Sense of Belonging was assessed using a 10-item scale. The questions were adapted from Rovai’s 20-item scale focused on measuring classroom community [88]. The ten items used in this study focused on connectedness. Students were prompted to think about the program and asked, for example, whether they feel “the students care about each other,” “I trust other students,” “isolated,” or “other students depend on me” (see S1 Appendix all measure items and response options). Responses were coded “strongly disagree” = 1, “disagree” = 2, “neutral” = 3, and “agree” = 4. Composite scores were created by averaging item responses to form a single composite score. Composite scores were calculated for pre-test and post-test items. Composite scores were calculated for pre-test items (α = 0.879) and post-test items (α = 0.884).

#### Mentor relationship

Students’ relationship with their mentor was assessed using a 9-item scale. The questions were adapted from Rovai’s 20-item scale [88]. The ten items used in this study focused on the connectedness between the student and mentor. Students were prompted to think about their mentor and asked, for example, whether they feel “connected to my mentor,” “isolated,” or “confident that my mentor will support me” (see S1 Appendix all measure items and response options). Responses were coded “strongly disagree” = 1, “disagree” = 2, “neutral” = 3, and “agree” = 4. Composite scores were created by averaging item responses. Composite scores were calculated for pre-test and post-test items. Composite scores were calculated for pre-test items (α = 0.871) and post-test items (α = 0.904).

#### Mentee Knowledge

The student’s understanding of their responsibilities as a mentee was assessed with an 8-item scale. The measure was created by research team members using the wording from the mentor-mentee contract, a form they completed before the Summer JumpStart program. Students were asked if they knew, for example, “how many hours I will work on [their] project with [their] mentor,” “what I must prepare to present to my mentor prior to our meetings,” and “what my mentor expects of me” (see Supplemental File for all items). Responses were coded “strongly disagree” = 1, “disagree” = 2, “neutral” = 3, and “agree” = 4. Composite scores were created by averaging item responses. Composite scores were calculated for pre-test and post-test items. Composite scores were calculated for pre-test items (α = 0.897) and post-test items (α = 0.924).

#### Health Assessment

The measure was created by research team members based on the BUILD PODER’s SJS Health and Well-being curriculum. For example, students were asked how often they do the following, “exercise or go to the gym,” “get enough sleep,” “make time for self-reflection, prayer or meditation,” and “stay in contact with important people in your life” (see Supplemental File for all items). Responses were coded “it will probably never occur to me” = 1, “never” = 2, “rarely” = 3, “sometimes” = 4, and “frequently” = 5. Composite scores were created by averaging item responses. Composite scores were calculated for pre-test items (α = 0.744) and post-test items (α = 0.826).

#### Stress Management

Student Stress Management was assessed using one item. Students were asked to “rate their ability to manage the overall level of stress they experience.” Responses were coded as “poor” = 1, “fair” = 2, “neutral” = 3, “good” = 4, and “excellent” = 5, and the research team created the measure.

#### Student satisfaction

Student satisfaction with the Summer JumpStart program was assessed using a 7-item measure adapted from the Client Satisfaction Questionnaire (CSQ-8) [89], which was divided into three subscales (Satisfaction 1, Satisfaction 2, and Satisfaction 3) (see S1 Appendix all measure items and response options). Averaging item responses to each question created composite scores. Students were only asked about student satisfaction in the post-test survey. Therefore, composite scores were calculated for post-test items only. Cronbach’s alpha statistics were computed for Satisfaction 1 (α = 0.852), Satisfaction 2 (α = 0.724), and Satisfaction 3 (α = 0.867) subscales.

#### Analysis strategy

Participant demographics for the analytic sample (n = 92) were calculated by cohort (Table 1). The average mean and standard deviation were calculated for each measure (pre-test and post-test) by cohort (Table 2). We conducted a one-way ANOVA analysis and Tukey HSD post hoc tests to examine how student satisfaction scores differed by cohort. To examine Cohort (i.e., 2019, 2020, 2021) and Time (i.e., pre-test versus post-test) and interaction of Cohort and Time for Research Self-Efficacy, Sense of Belonging, Mentor Relationship, Mentee Knowledge, Health Assessment, and Stress Management measures, we conducted a two-way mixed ANOVA analysis and Tukey HSD post hoc tests. Due to less than 5% missing data, participants with missing data on any measure used in the analysis were removed from the sample [90]. Therefore, the final analytic sample consisted of 92 participants, with one participant in 2021 removed due to not answering mentor-related questions in the post-test survey. Statistical analyses were performed using Stata (version 17) and SPSS (version 28.0).

**Table 1:** Participant characteristics by cohort.

Characteristic	2019	2020	2021	Total
**N**	26	33	33	92
	**N (%)**	**N (%)**	**N (%)**	**N (%)**
**Gender**				
Female	21 (80.77)	20 (60.61)	25 (75.75)	66 (71.73)
Male	5 (19.23)	13 (39.39)	8 (24.24)	26 (28.26)
**Ethnicity/Race**				
Asian	4 (15.38)	6 (18.18)	5 (15.15)	15 (16.3)
Black	-	-	6 (18.18)	6 (6.52)
Latinx	11 (42.31)	23 (69.70)	16 (48.48)	50 (54.34)
White (non-Hispanic)	6 (23.08)	3 (9.09)	3 (9.09)	12 (13.04)
Other	1 (3.85)	1 (3.03)	3 (9.09)	5 (5.43)
**First Generation student**	12 (46.15)	22 (66.67)	22 (66.66)	56 (60.86)
**Pell Grant recipient**	13 (50)	20 (60.61)	13 (39.39)	46 (50)
**Major affiliated college**				
Health and Human Development	4 (15.38)	5 (15.15)	3 (9.09)	12 (13.04)
Social and Behavioral Science	10 (38.46)	10 (30.30)	16 (48.48	36 (39.13)
Science and Mathematics	11(42.3)	15 (45.45)	8 (24.24)	34 (36.95)
Engineering and Computer Science	1 (3.84)	3 (9.09)	6 (18.18)	10 (10.86)

**Table 2:** Descriptive statistics for study measures (pre-test and post-test) by cohort.

Measure	2019 (n = 26)		2020 (n = 33)		2021 (n = 33)	
	*M*	*SD*	*M*	*SD*	*M*	*SD*
**Research self-efficacy**						
pre-test	3.76	0.646	3.64	0.618	3.71	0.660
post-test	4.39	0.518	4.21	0.557	4.22	0.613
**Sense of Belonging**						
pre-test	3.21	0.239	3.13	0.314	3.15	0.270
post-test	3.63	0.231	3.41	0.259	3.65	0.380
**Mentor Relationship**						
pre-test	3.00	0.249	2.96	.219	2.94	0.306
post-test	3.10	0.199	3.03	.247	3.11	0.274
**Mentee Knowledge**						
pre-test	3.76	0.737	3.61	.757	3.47	0.731
post-test	4.01	0.628	3.84	.668	3.76	0.618
**Health**						
pre-test	3.89	0.273	3.90	0.443	3.95	0.377
post-test	4.53	0.283	4.28	0.394	4.22	0.404
**Stress Management**						
pre-test	3.26	0.961	3.27	1.00	3.15	1.06
post-test	4.11	0.816	4.06	0.658	3.73	1.00
**Student Satisfaction (post-test)**						
Satisfaction 1	4.52	0.590	4.40	0.569	4.54	0.549
Satisfaction 2	4.78	0.428	4.80	0.373	4.92	0.217
Satisfaction 3	4.65	0.524	4.51	0.522	4.77	0.463

#### Ethics statement

All participants provided informed written consent online before completing any surveys for this study. This study was approved by the CSUN Committee for the Protection of Human Subjects under IRB-FY21-222.

## Results

Among the participants, 71.73% identified as female and 28.26% as male (Table 1). Among the participants, 54.34% were Latinx, 16.3% were Asian, 13.04% were White, 6.52% were Black, 5.43% were Other (multi-ethnic), 60.86% were first-generation, and 50% were Federal Pell Grant recipients. Among the participants, 13.04% identified as health and human development majors (e.g., public health, nutrition), 39.13% identified as social and behavioral science majors (e.g., psychology), 36.95% identified as science and mathematics majors (e.g., biology, chemistry), and 10.86% identified as engineering and computer science majors.

### Research Self Efficacy

There was a significant main effect of Time on Research Self-Efficacy scores overall (*F*(1,89) = 61.29, *p* < .001, η_p_^2^ = 0.408), such that scores increased significantly from pre-test to post-test among participants in all cohorts (see Table 2 for means and standard deviations of all measures). However, the effect for Cohort was not significant (*F*(2,89) = 0.67, p = 0.512, η_p_^2^ = 0.015), and the interaction between Time and Cohort on Research Self-Efficacy scores was not significant (*F*(2,89) = 0.22, *p* = 0.807, η_p_^2^ = 0.005).

### Sense of Belonging

There was a significant main effect of Time on Sense of Belonging scores overall (*F*(1,89) = 170.123, *p* < 0.001, η_p_^2^ = 0.657), such that average scores increased significantly from pre-test to post-test among participants in all cohorts. There was also a significant Cohort effect on Sense of Belonging scores (*F*(2,89) = 4.158, *p* = 0.019, η_p_^2^ = 0.085). Tukey post hoc tests show scores in the 2019 cohort (i.e., entirely in-person) were significantly higher (0.328 mean difference) than in the 2020 cohort (i.e., entirely online) at *p* < .05, but no other difference was significant. There was no significant interaction between Time and Cohort on Sense of Belonging scores (*F*(2,89) = .56, *p* = 0.571, η_p_^2^ = 0.013).

### Mentor Relationship

There was a significant main effect of Time on Mentor Relationship scores overall (*F*(1,89) = 69.86, p < 0.001, η_p_^2^ = 0.440), such that scores increased significantly from pre-test to post-test among participants in all cohorts. However, the main effect of Cohort was not significant (*F*(2,89) = 2.44, *p* = 0.093, η_p_^2^ = 0.052), and the interaction between Time and Cohort on Mentor Relationship scores was also not significant (*F*(2,89) = 1.78, *p* = 0.17, η_p_^2^ = 0.038).

### Mentee Knowledge

There was a significant main effect of Time on Mentee Knowledge scores overall (*F*(1,89) = 79.06, *p* < 0.001, η_p_^2^ = 0.470), such that scores increased significantly from pre-test to post-test among participants in all cohorts. However, the main effect of Cohort was not significant (*F*(2,89) = 1.17, *p* = 0.315, η_p_^2^ = 0.026), and the interaction between Time and Cohort on Mentee Knowledge scores was also not significant (*F*(2,89) = 0.352, *p* = 0.704, η_p_^2^ = 0.008).

### Health Assessment

There was a significant main effect of Time on Health Assessment scores overall (*F*(1,89) = 104.57, *p* < 0.001, η_p_^2^ = 0.540), such that scores increased significantly from pre-test to post-test among participants in all cohorts. The main effect of Cohort on Health Assessment scores was not significant (*F*(2,89) = 1.33, p = 0.267, η_p_^2^ = 0.029). However, there was a significant interaction between Time and Cohort on Health Assessment scores (*F*(2,89) = 6.34, *p* = .03, n_p_^2^ = 0.125), indicating that the scores in the 2019 cohort increased at a significantly faster rate than observed in the 2020 and 2021 cohorts.

### Stress Management

There was a significant main effect of Time on Stress Management scores overall (*F*(1,89) = 37.50, *p* < .001, η_p_^2^ = .296), indicating that students in all cohorts significantly increased their ability to manage stress from pre-test to post-test. However, the main effect of Cohort was not significant (*F*(2,89) = 1.12, *p* = .331, η_p_^2^ = .025), and the interaction between Time and Cohort on Stress Management scores was not significant (*F*(2,89) = .474, p = .624, η_p_^2^ = .011).

### Student satisfaction

There was no statistically significant difference between the average Satisfaction 1 score by Cohort (*F*(2,90) = 0.612, p = .545, η_p_^2^ = .013). There was no statistically significant difference between average Satisfaction 2 scores by Cohort (*F*(2,90) = 1.55, p = .217, η_p_^2^ = .033). There was no statistically significant difference between average Satisfaction 3 scores by Cohort (*F*(2,90) = 2.31, p = .104, η_p_^2^ = .049).

## Discussion

Findings from this study highlight URE student outcomes over time and across different platforms (in-person pre-pandemic, remote during COVID-19 quarantine, and hybrid) by comparing student surveys conducted in 2019, 2020, and 2021. This study found that despite the change in platforms, student scores across the three cohorts increased significantly from pre-test to post-test for Research Self-Efficacy, Sense of Belonging, Mentor Relationship, Mentee Knowledge, Health Assessment, and Stress Management. In addition, there were no significant differences between program satisfaction scores by year. Our findings indicate that URE programs implemented remotely and using a hybrid format can provide students with an effective and impactful URE experience.

During the summer of 2020, several UREs pivoted from in-person to remote online training in response to the COVID-19 pandemic [7, 10–12]. Of these programs, three studies examined the efficacy of remote online training by measuring student and program outcomes after training was completed [11–13], and only one study compared pre-pandemic URE program implementation to pandemic program implementation [14]. The current study built on previous research by measuring URE student outcomes across three different types of program implementation in 2019, 2020, and 2021. Examining student scores across different URE program design and implementation strategies is critical. The current study provides program design recommendations to URE principal investigators (PIs), faculty, and staff who may need to shift in-person URE programming to remote or hybrid program models, especially UREs serving historically minoritized groups [7].

The current study found that despite the change in URE platforms, student Research Self-Efficacy scores increased significantly from pre-test to post-test. These results indicate that participants felt more confident engaging with research activities by the end of the SJS program. These findings parallel a study by Ajayi and colleagues, who compared student outcomes before and during the COVID quarantine [14]. Ajayi and colleagues found that students in the 2019 and 2020 URE programs reported significant gains in confidence conducting research [14]. Moreover, previous studies used a pre-test and post-test design in 2020 to examine remote URE programs and found that remote URE participants reported gains in research self-efficacy [12, 83].

It is well established that traditional in-person UREs can increase research self-efficacy among undergraduates [34, 64, 65]. The current study builds on these findings by showing that online UREs can also increase research self-efficacy among undergraduates. In the current study, students in the SJS program were matched with research mentors and received research training in person and remotely. Our results are notable because they suggest that remote UREs can also support persistence in STEM and encourage undergraduates to pursue careers in research [61, 63].

The current study also found that student Sense of Belonging scores increased significantly from pre-test to post-test. However, the 2019 (in-person) cohort scores were significantly higher than the 2020 (online) cohort scores. These findings are consistent with past research, which found that students felt that online UREs could improve outcomes by focusing on cohort building programming and activities [7]. Differences in Sense of Belonging may be influenced by cohort characteristics and/or the program platform. For example, the 2020 cohort was the first wave of students required to shift from in-person to remote learning, therefore students in this cohort may not have found online socialization as impactful as in-person socialization, or students needed time to adjust to informal interactions with peers online.

Findings from previous research indicate that remote URE participants can develop a sense of belonging [14, 83, 91]. A study by Alaee and colleagues noted that remote URE participants experienced a sense of belonging by communicating and socializing with lab mates and other program participants [83]. Additionally, intimate conversations and whole-group discussions help remote URE participants feel connected with each other [7, 12]. Sense of Belonging is critical, as it greatly affects emotional and cognitive functioning [85]. In the current study, activities to build relationships with peers and program staff were implemented in-person or remotely in 2019, 2020, and 2021. Program leadership implemented weekly or monthly events for all students in the program so participants could socialize and interact with their cohort and the larger group. In addition, across all cohorts and platforms, SJS students participated in team-building activities, movie nights, and tours of local museums or communities (in-person or online). Virtual engagement has been shown to foster feelings of belonging in historically minoritized STEM female faculty [92]. It is worth noting that Sense of Belonging in the current study captured participants’ growth in feelings of acceptance by their research team. Future research should examine how URE participants develop feelings of acceptance by the broader scientific research community.

The current study found that student Mentor Relationship and Mentee Knowledge scores increased significantly from pre-test to post-test. These results are consistent with past research indicating that remote UREs can complement traditional forms of mentorship [10] and that mentees completing remote UREs can have meaningful and productive virtual mentoring experiences [12, 93]. A growing body of research demonstrates that virtual mentoring can facilitate social, academic, and career success and enables the development of transferable and technical skills similar to the benefits of in-person mentoring [94–100]. In the current study, all SJS program participants had multiple opportunities to interact with their mentors, potentially affecting their mentoring relationships positively. Moreover, all participants were required to submit a mentor-mentee contract, establishing clear expectations at the program’s start. These program components were required of in-person and remote participants, which may explain why SJS mentees perceived their mentoring relationships favorably.

The results from the current study and previous research indicate that remote mentorship offers an ability to continue training in STEM research and promotes feelings of interpersonal connection. Further, remote mentoring can also have added benefits, such as increased flexibility in meeting times/locations and a more comfortable environment for mentee communication [97, 101, 102]. These benefits may be essential to historically minoritized students with financial or familial obligations. Future studies will be necessary to determine the long-term effects of remote research mentorship, such as job placement and retention in STEM.

The current study found that student Health Assessment and Stress Management scores increased significantly from pre-test to post-test. The results indicate that students felt more capable of managing their health and stress by the end of the program. These findings parallel past research which involved 170 remote URE participants in the summer of 2020 and found that participants reported gains in research self-efficacy and shifts in mental well-being. For example, participants reported decreased stress, resilience gains, and improved life satisfaction [12]. The results from the current study suggest that SJS sessions focused on stress and physical were impactful. The SJS program provided students with the tools to decrease stress and improve resilience and mitigate burnout [103–105]. Furthermore, undergraduate researchers’ shifts in mental well-being, such as improved life satisfaction, promote STEM excellence in a wellness environment [106].

### Limitations

The study was limited to college students in Southern California. Therefore, results may not be generalizable to college students living in other regions of the United States. Most participants in the current study were female, lower-income, and first-generation college students, which may limit the generalizability of findings to other sociodemographic groups. In addition, some academic disciplines may be better adapted to an online URE experience than others. The BUILD PODER online URE was designed for students majoring in science and mathematics and social and behavioral sciences, which limits the generalizability of this program to other academic disciplines.

## Conclusion

The results from the current study demonstrate that the core objectives of traditional in-person UREs can be accomplished virtually while also impacting students’ well-being. In addition, these results may provide added benefits compared to in-person programs. For instance, remote UREs could engage more historically minoritized students, who may experience barriers to access, such as work commitments, financial constraints, geographic limitations, childcare, or family obligations. As the scientific research community confronts its lack of diversity, remote experiences could be leveraged to address existing inequities. Altogether, the results indicate that remote URE programs afford many of the same opportunities and benefits as in-person programs. Virtual programming can target and improve the student well-being necessary for academic, career, and life satisfaction and success, bolstering science pathways for all students. Future studies should investigate how to scale up remote URE offerings and examine whether remote URE participants pursue graduate education and research-related careers at the same rates as in-person program participants.

## Supporting information

S1 Fig URE recruitment & program timeline. (PDF)

S1 Appendix JumpStart study measures. (DOCX)

S1 Dataset Jumpstart minimal data set deidentified. (SAV)
