# Supplementary figures and images for: Comparing undergraduate research experiences before, during, and after the COVID-19 quarantine: The successful adaptation of the BUILD PODER Summer JumpStart program
Source: PLoS One. 2023 Dec 28;18(12):e0295901. doi: 10.1371/journal.pone.0295901 (PMC10754433; doi:10.1371/journal.pone.0295901)

# Student Training Recruitment

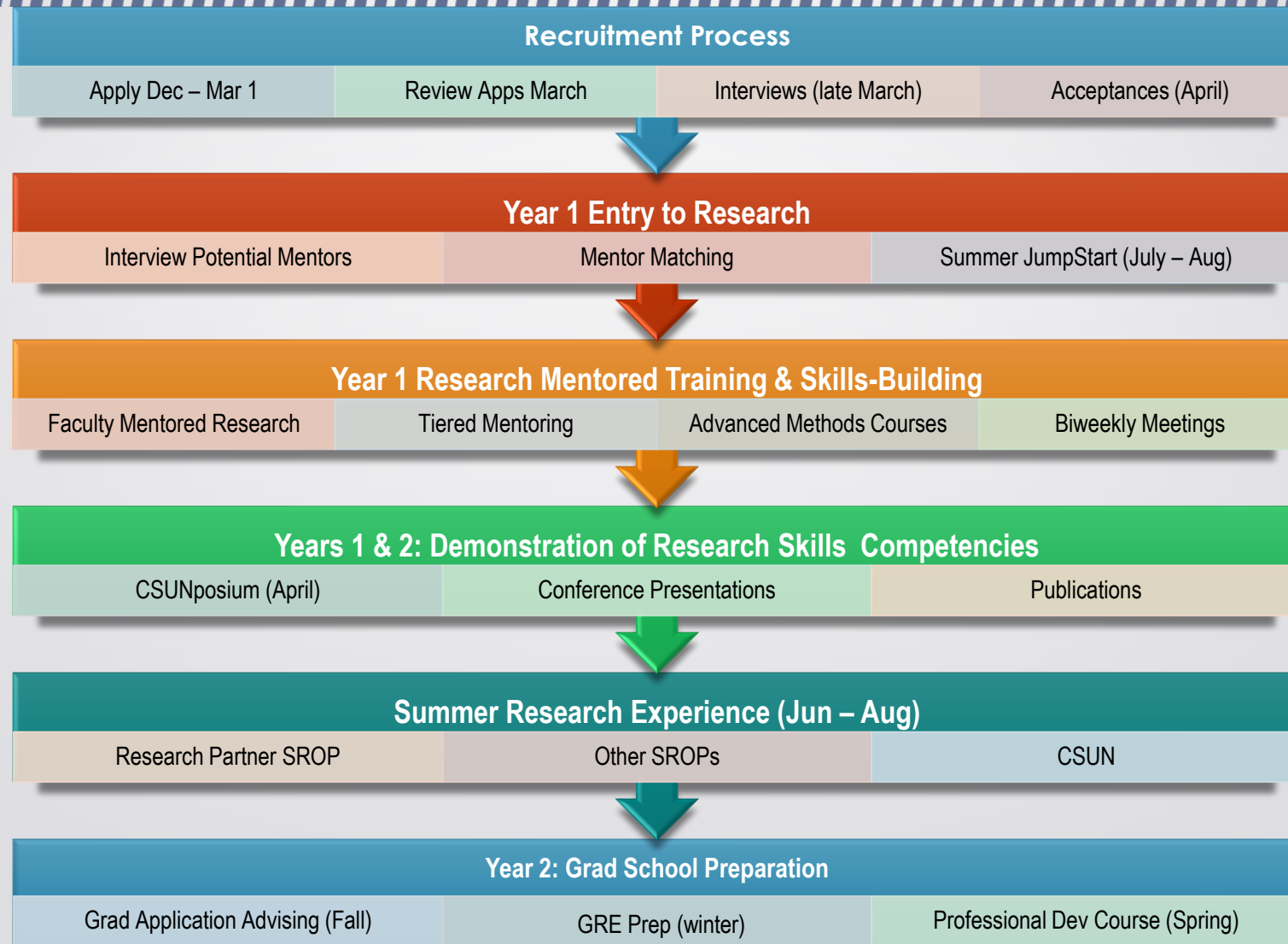

Supplement: S1 Fig — (PDF) [file pone.0295901.s001.pdf]
