# Supplementary material for: Comparing undergraduate research experiences before, during, and after the COVID-19 quarantine: The successful adaptation of the BUILD PODER Summer JumpStart program
Source: PLoS One. 2023 Dec 28;18(12):e0295901. doi: 10.1371/journal.pone.0295901 (PMC10754433; doi:10.1371/journal.pone.0295901)
Supplement: S1 Appendix — (DOCX) [file pone.0295901.s002.docx]

**Supporting Information**

**Methods: Summer JumpStart Study Measures**

| Measure**: Sense of Belonging** | |
| --- | --- |
|  | Please indicate how much you agree or disagree with the following statements. When I think about the BUILD PODER program, I feel…  1 = “Strongly Disagree”  2 = “Disagree”  3 = “Neutral”  4 = “Agree”  5 = “Strongly Agree” |
| Community_1 | - the students care about each other. |
| Community_2 | - connected to other students. |
| Community_3 | - there is no spirit of community. |
| Community_4 | - this program is like a family. |
| Community_5 | … - isolated. |
| Community_6 | - I trust other students. |
| Community_7 | - I can rely on other students. |
| Community_8 | - other students depend on me. |
| Community_9 | - uncertain about other students. |
| Community_10 | - confident that other students will support me. |

| Measure**: Mentoring Relationship** | |
| --- | --- |
|  | Please indicate how much you agree or disagree with the following statements. When I think about my BUILD PODER faculty mentor, I feel…  1 = “Strongly Disagree”  2 = “Disagree”  3 = “Neutral”  4 = “Agree”  5 = “Strongly Agree” |
| Mentor_1 | - my mentor cares about me. |
| Mentor_2 | - connected to my mentor. |
| Mentor_3 | - isolated. |
| Mentor_4 | - I don’t trust my mentor. |
| Mentor_5 | - my mentor doesn’t trust me. |
| Mentor_6 | - I can rely on my mentor. |
| Mentor_7 | - my mentor depends on me. |
| Mentor_8 | - uncertain about my mentor. |
| Mentor_9 | - confident that my mentor will support me. |

| Measure**: Mentee Knowledge** | |
| --- | --- |
|  | Please indicate how much you agree or disagree with the following statements. When thinking about my responsibilities as a BUILD PODER mentee, I know…  1 = “Strongly Disagree”  2 = “Disagree”  3 = “Neutral”  4 = “Agree”  5 = “Strongly Agree” |
| Mentee_1 | - how many hours I will work on my project with my mentor during the academic year. |
| Mentee_2 | - what the primary means of communication with my mentor will be. |
| Mentee_3 | - whose responsibility it is to schedule meetings with my mentor. |
| Mentee_4 | - what I must prepare to present to my mentor prior to our meetings. |
| Mentee_5 | - how my mentor will provide feedback regarding my performance during our meetings. |
| Mentee_6 | - the procedures my mentor will take in training me during our meetings (e.g. writing out directions, and handouts) |
| Mentee_7 | - how to document research results to present to my mentor. |
| Mentee_8 | - what my mentor expects of me. |

| Measure: **Research Self-efficacy** | |
| --- | --- |
|  | Please rate your level of confidence or comfort with the following research activities:  1 = “None”  2 = “Little”  3 = “Moderate”  4 = “Quite a Bit”  5 = “A Lot” |
| Activities_1 | - Confidence in my ability to contribute to science. |
| Activities_2 | - Comfort in discussing scientific concepts with others. |
| Activities_3 | - Confidence in my ability to do well in future science courses. |
| Activities_4 | - Ability to work independently. |
| Activities_5 | - Developing patience with the slow pace of research. |
| Activities_6 | - Understanding what everyday research work is like. |
| Activities_7 | - Taking greater care in conducting procedures in the lab or field. |
| Activities_8 | - Confidence in engaging in real-world science research. |
| Activities_9 | - Comfort in working collaboratively with others. |

| Measure: **Health Assessment** | |
| --- | --- |
|  | How often do you do each of the following...  1 = “It will probably never occur to me”  2 = “Never”  3 = “Rarely”  4 = “Sometimes”  5 = “Frequently” |
| Health1_1 | Eat regularly (e.g. breakfast & lunch) |
| Health1_2 | Eat healthfully |
| Health1_3 | Exercise, or go to the gym |
| Health1_4 | Get regular medical care for prevention |
| Health1_5 | Get medical care when needed |
| Health1_6 | Take time off when you're sick |
| Health1_7 | Get enough sleep |
| Health1_8 | Take a step to decrease stress in your life |
| Health1_9 | Express your outrage in a constructive way |
| Health2_1 | Get away from stressful technology such as cellphones, e-mail, social media |
| Health2_2 | Make time for self-reflection, prayer, meditation |
| Health2_3 | Write in a journal |
| Health2_4 | Read literature unrelated to school |
| Health2_5 | Spend time outdoors |
| Health2_6 | Engage your intelligence in a new area - go to an art museum, performance, sports event, exhibit, etc. |
| Health2_7 | Spend time with others whose company you enjoy |
| Health2_8 | Stay in contact with important people in your life |
| Health2_9 | Treat yourself kindly (supportive inner dialogue/self-talk) |
| Health2_10 | Express gratitude |

| Measure: **Stress** (1 item) | |
| --- | --- |
| Stress | Within the last 12 months, how would you rate your ability to manage the overall level of stress you experience?  1 = “Poor”  2 = “Fair”  3 = “Neutral”  4 = “Good”  5 = “Excellent” |

| Measure: **Student Satisfaction** | |
| --- | --- |
| 1 = “Not at all”  2 = “A Little”  3 = “Somewhat”  4 = “Very Much”  5 = “Definitely” | |
| satisf1_1_p | Did you get the kind of training you wanted? |
| satisf1_2_p | Did the program meet your needs? |
| satisf1_3_p | Did the training you received help you to be a more successful student? |
|  | |
| 1 = “Extremely unlikely”  2 = “Somewhat unlikely”  3 = “Neither likely nor unlikely”  4 = “Somewhat likely”  5 = “Extremely likely” | |
| satisf2_1_p | If a friend were in need of similar help, how likely would you recommend the program to him or her? |
| satisf2_2_p | If you were to seek training again, how likely would you come back to this program? |
|  | |
| 1 = “Very Dissatisfied”  2 = “Dissatisfied”  3 = “Neutral”  4 = “Satisfied”  5 = “Very Satisfied” | |
| satisf3_1_p | Overall, how satisfied are you with the training you received? |
| satisf3_2_p | Overall, how satisfied are you with the quality of the JumpStart workshop? |
